# Genome-Centric Dynamics Shape the Diversity of Oral Bacterial Populations

**DOI:** 10.1128/mbio.02414-22

**Published:** 2022-10-10

**Authors:** Daniel R. Utter, Colleen M. Cavanaugh, Gary G. Borisy

**Affiliations:** a Department of Organismic and Evolutionary Biology, Harvard University, Cambridge, Massachusetts, USA; b The Forsyth Institute, Cambridge, Massachusetts, USA; Baylor College of Medicine

**Keywords:** metagenomics, microbial evolution, oral microbiome, strain dynamics, time series

## Abstract

Two major viewpoints have been put forward for how microbial populations change, differing in whether adaptation is driven principally by gene-centric or genome-centric processes. Longitudinal sampling at microbially relevant timescales, i.e., days to weeks, is critical for distinguishing these mechanisms. Because of its significance for both microbial ecology and human health and its accessibility and high level of curation, we used the oral microbiota to study bacterial intrapopulation genome dynamics. Metagenomes were generated by shotgun sequencing of total community DNA from the healthy tongues of 17 volunteers at four to seven time points obtained over intervals of days to weeks. We obtained 390 high-quality metagenome-assembled genomes (MAGs) defining population genomes from 55 genera. The vast majority of genes in each MAG were tightly linked over the 2-week sampling window, indicating that the majority of the population’s genomes were temporally stable at the MAG level. MAG-defined populations were composed of up to 5 strains, as determined by single-nucleotide-variant frequencies. Although most were stable over time, individual strains carrying over 100 distinct genes that rose from low abundance to dominance in a population over a period of days were detected. These results indicate a genome-wide as opposed to a gene-level process of population change. We infer that genome-wide selection of ecotypes is the dominant mode of adaptation in the oral populations over short timescales.

## INTRODUCTION

The means by which bacterial populations change within complex communities remain central issues in microbial ecology. Broadly, genetic change within microbial populations can be conceptualized as occurring through gene-centric or genome-centric processes or some combination thereof. Gene-centric processes like recombination and gene duplication allow individual genes or gene clusters to change in frequency as they sweep through the population more or less independently (1–5). Genome-centric processes involve selection across numerous functionally distinct yet tightly linked loci within a genome, thus changing the frequency of an entire genome or set of related genomes (6–8). While both processes are at work in most microbial systems, the relative contribution of each process can engender different perspectives of microbial ecology and evolution. For example, the “ecotype” hypothesis, which views microbial populations as composed of different ecologically distinct subtypes with their own distinct genomes, weighs genome-centric processes heavily.

Distinguishing gene- versus genome-centric processes in a complex natural microbiome necessitates analyzing genomes representative of the members living in that microbiome. Metagenomes, resulting from the shotgun sequencing of total DNA from a sample, capture a snapshot of the genomic composition of a community yet are fragmented short reads lacking genomic context (9). Metagenome-assembled genomes (MAGs) avoid cultivation bias and so can represent the genomes of *in situ* microbial populations with accuracy, although MAGs are typically limited by fragmentation and lack of completeness (10, 11). Recent studies have generated hundreds of thousands of MAGs from published human metagenomes, underscoring the extent of genomic diversity within the human microbiome (10, 12). However, most of these metagenomes present single- or dual-time-point snapshots; thus, questions remain about how the genetic composition of a population varies over time.

Longitudinal sampling at microbially relevant timescales is critical to distinguishing gene-centric versus genome-centric dynamics (8). While both processes produce genetic change in a population, they differ in the predicted dynamics and timescales. Gene-centric adaptation predicts incremental change through gene-specific sweeps (13), while genome-centric adaptation predicts more drastic changes through preexisting genomes sweeping to abundance (14). Thus, the two processes can be distinguished by observing whether populations change through gene-specific changes or by groups of genes changing together. Implicit in this approach is that the process must be monitored at timescales relevant to microbial growth and selection.

Longitudinal sampling is also methodologically advantageous, as it can improve confidence in the quality of MAGs that are generated (9). Since the same population is typically resampled over a time series, the process of binning assembled contiguous fragments (contigs) into MAGs is improved by the expectation that all parts of the same chromosome should have high correlation (15, 16). This expectation also allows a means of quality control by identifying misplaced contigs based on aberrant coverage relative to other MAGs (16).

Microbial dynamics in complex microbiomes have been studied by a variety of sequence methods, from marker gene sequencing to metagenome analysis to MAG construction, generally focusing on the gut microbiome and at long timescales (months to years). Examples of intraspecies phase shifts and habitat restriction were discovered via 16S rRNA amplicon sequence variants (ASVs) in the human microbiome and presumed to reflect different ecotypes (17–21). Sharon et al. sequenced infant gut metagenomes and identified rapid changes among populations on the order of days (22), and further analyses of the same data revealed changing single-nucleotide-polymorphism (SNP) frequencies within those fluctuating populations (23). Zhao et al. (24) cultivated and sequenced numerous Bacteroides fragilis isolate genomes in conjunction with metagenomic sequencing to reveal that each volunteer’s gut B. fragilis population was dominated by a few strains differing by single-digit numbers of SNPs. Notably, they found strain coalescence within approximately 1 year. Garud et al. (4) found genomic evolution within human gut populations, with mutational accumulation being the most relevant at monthly timescales while strain replacement dominated at longer timescales. In the high-selection environment of hospitals, Evans et al. (25) documented frequent (on the order of days to weeks) mobile element-mediated horizontal gene transfer between populations isolated from infections. In a long-term longitudinal survey of the gut microbiome of a single human, treatment with an antibiotic seemingly induced genome-wide sweeps within some populations (26). Outside the human microbiome, serial passaging of laboratory communities derived from pitcher plant phytotelmata revealed different ecological dynamics among genomically related strains (27). These reports emphasize different processes of microbial adaptation in complex microbiomes at various timescales, chiefly focusing on the human gut microbiome.

The human oral microbiome represents a distinct microbial environment from the gut and a readily accessible site for investigating basic questions of microbial ecology. Approximately 700 recognized bacterial species inhabit the oral cavity (28), the majority of which display sharply differential abundances among the different oral sites (10), leading to the hypothesis that the majority of oral bacterial populations are specialists adapted to a specific oral habitat (29). In contrast, the gut microbiota is thought to harbor thousands of species (30, 31). Further, oral microbes typically exist in highly ordered, discrete biofilms (32–34), in contrast to the better-mixed gut bacteria (35, 36). Combined with the mouth’s direct exposure to exogenous factors, these ecological distinctions of the oral microbiome could result in intrapopulation dynamics different from those elsewhere in the human microbiome.

These considerations of adaptation emphasize the importance of matching the timescale of analysis to the biology of the microbiome. Thus, we collected a metagenomic time series from the healthy human tongue dorsa of 17 volunteers, sampled over a period of days to 2 weeks, to investigate changes in microbial populations. Here, we use “population” to refer to a set of phylogenetically related bacteria defined and represented by a MAG. We constructed MAGs and tracked the abundance of genes and nucleotide variants within the MAGs to characterize gene- and genome-level dynamics. We conclude that intrapopulation adaptation in the oral microbiome over short timescales occurs primarily by genome-centric processes (i.e., replacement of subpopulations).

## RESULTS

### Assembly of 390 MAGs allows investigation of the population dynamics of oral bacteria.

Longitudinal studies using metagenome-assembled genomes (MAGs) allow the investigation of the genomic dynamics of microbial populations at unprecedented resolution. However, to have utility, the MAGs must meet certain criteria. First, they must be consistently detected over time; second, the MAGs must be faithful representations of natural microbial populations. Therefore, attention must be paid to the quality of the MAGs generated. Ideally, these MAGs would sample many diverse oral taxa; however, for the purposes of this study, all taxa need not be represented, as our focus was on generating high-quality MAGs to serve as indicators for the genome dynamics of microbial populations within individuals.

We generated 390 unique MAGs from 81 tongue samples obtained from 17 volunteers sampled at 4 to 7 time points over a 2-week period. Contiguous stretches of nucleotides (contigs) were assembled in a single step for each donor using all of that donor’s time points and binned with CONCOCT (15) to generate rough genome bins. Bins were manually refined in anvi’o (23) using differential coverage, tetranucleotide frequency, and SNV frequencies to remove misplaced contigs (9, 16, 37) (see Materials and Methods). Taxonomic annotation according to the Genome Taxonomy Database (38) assigned the MAGs to 55 genera, including the common oral genera *Prevotella*, *Actinomyces*, and *Porphyromonas* (see Fig. S1 and Table S1 in the supplemental material). *Prevotella* was the most represented genus among the data set with 77 MAGs (Fig. S1). Other commonly abundant genera were represented by fewer MAGs, likely due to assembly artifacts (see Materials and Methods). These 390 MAGs form the basis of our analyses into the population and subpopulation dynamics of the oral microbiome.

10.1128/mbio.02414-22.1 TABLE S1 Taxonomic assignment for each MAG along with summary metrics (length in base pairs, estimated completion, and redundancy). The second tab (“Detection”) reports the fraction of each MAG recruiting any coverage for all samples, and the third tab (“Coverage”) reports the coverage of each MAG in each sample. Download Table S1, XLSX file, 0.9 MB. Copyright © 2022 Utter et al. 2022 Utter et al. https://creativecommons.org/licenses/by/4.0/ This content is distributed under the terms of the Creative Commons Attribution 4.0 International license.

10.1128/mbio.02414-22.3 FIG S1 Number of MAGs binned by genus. Download FIG S1, PDF file, 0.02 MB. Copyright © 2022 Utter et al. 2022 Utter et al. https://creativecommons.org/licenses/by/4.0/ This content is distributed under the terms of the Creative Commons Attribution 4.0 International license.

Operationally, we define a population as all the metagenomic reads that recruit to one MAG versus other MAGs. The recruiting MAG serves as the reference genome for that population. By tracking single nucleotide variants (SNVs) in core genes throughout a MAG across samples, we can identify which SNVs are tightly associated. From the tight associations between these core gene SNVs across samples, we infer that they are not shuffled by recombination and so mark different genomic variants. These distinct SNV combinations mark “haplotypes,” putative major genotypes within each population (39). These haplotypes represent subpopulations of a MAG-defined population, referred to here as strains.

### Many populations are consistently detected across multiple time points.

**FIG 1:**
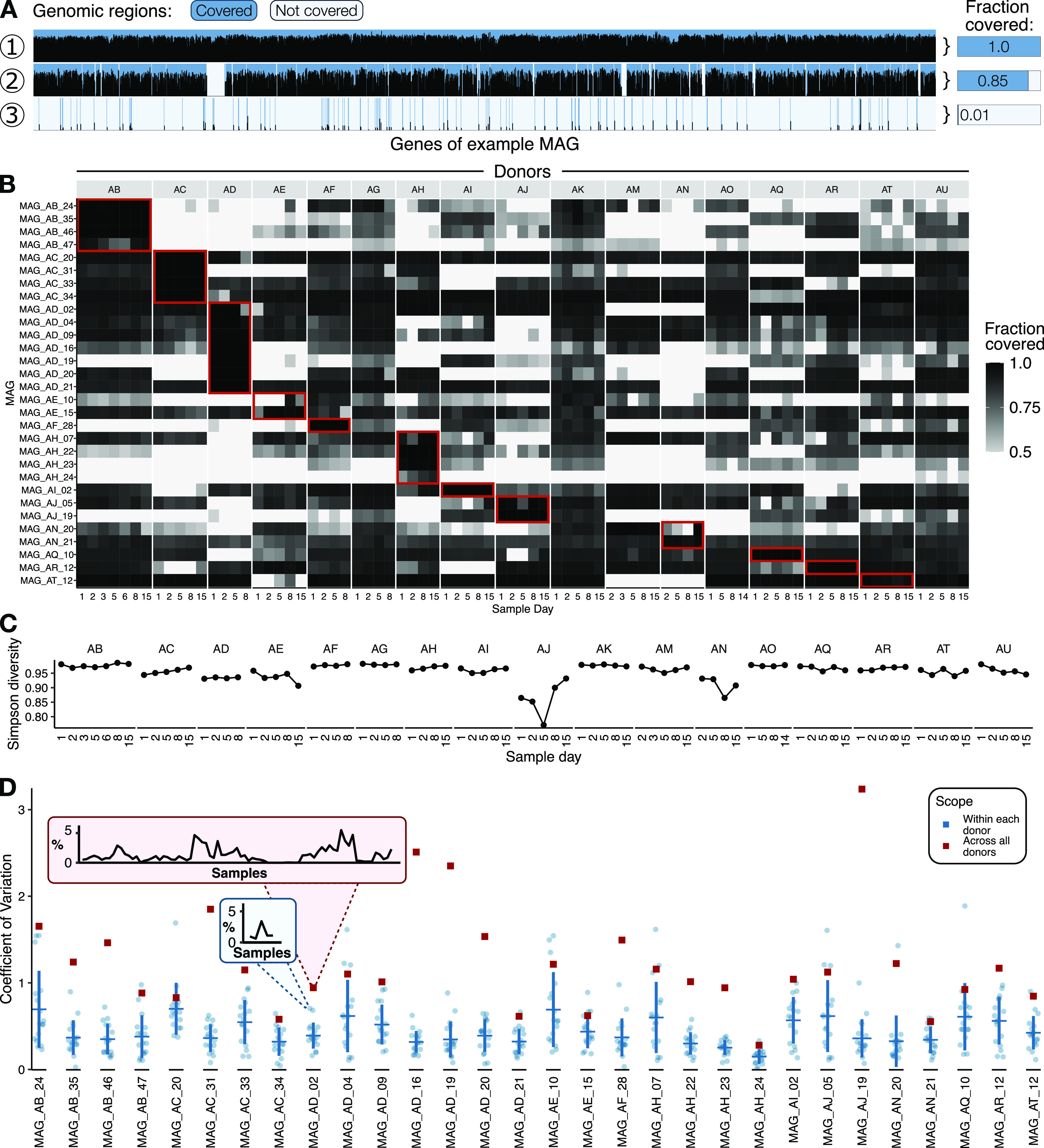
The 30 most abundant MAGs are detectable and stable over time within several individual mouths. (A) The fraction of a MAG covered in a sample provides a useful detection metric. Here, for each of three example metagenomes (tracks 1, 2, and 3), a cartoon shows the coverage received (height of thin black bars) for each gene (*x* axis) in a single MAG. The dark blue shading highlights regions receiving coverage from that metagenome, while the pale blue shading highlights regions not receiving coverage. The fraction covered (right) is calculated as the length of covered regions (dark blue) divided by the total length (dark blue + pale blue). (B) Many MAGs are detected across time points and in multiple donors. A heat map shows the fraction of the MAG covered at all (shading darkness) for the 30 MAGs with the highest average relative abundance. Each column shows the fraction detected in a sample, with samples arranged by donor (labeled at the top) in chronological order. Red boxes outline the samples from which each MAG was coassembled, revealing that many MAGs are detected outside their defining metagenomes. Only fractions of ≥0.5 are shown, since we consider MAGs with fewer than half of their nucleotides covered to not be confidently detected. (C) Simpson diversity of MAG relative abundances at each time point for each donor (groups of connected dots). (D) Coefficient of variation of MAG relative abundance for the 30 MAGs with the highest average relative abundance. For each MAG are plotted the coefficients of variation for that MAG within each of the 17 donors (blue dots) or across all samples from all donors (red dot). The red and blue inset boxes show the underlying relative abundance data behind two coefficient of variation calculations, one across samples from all donors (red) and within a single donor (blue). The horizontal and vertical bars show the mean coefficient of variation and 1 standard deviation, respectively.

To track the process of genetic change in a population, its representative MAG should be consistently detected across multiple time points, while subpopulations or gene frequencies may vary. The fraction of nucleotides in a MAG receiving any coverage provides a metric to assess a MAG’s presence in a sample (Fig. 1A). We consider a MAG to be detected in a sample if at least 50% of its genome is covered. With this criterion, the 30 MAGs receiving the highest mean percent coverage across all samples were detected consistently within individual subjects and across samples from multiple mouths (Fig. 1B; Fig. S2). Indeed, 241 MAGs were detected in 10 or more samples, and 83 MAGs were detected in at least 40 samples (Fig. S3); thus, the majority of MAGs were detected in at least two mouths across multiple samples, and approximately one-fifth of MAGs were detected in half the samples. Therefore, when the genome dynamics of populations (MAGs) are analyzed, the same population can be analyzed in multiple donors, avoiding any possible biases associated with inferences based solely on samples from which a MAG is defined.

10.1128/mbio.02414-22.4 FIG S2 (A) Fraction of MAG covered at all (shading intensity) for all 390 MAGs (rows), with the rows arranged to cluster MAGs with similar fraction covered in each metagenome (columns). Each column shows an individual sample, grouped by donor. Only fractions of ≥0.5 are shown, since we consider MAGs with fewer than half of their nucleotides covered to not be confidently detected. Red boxes mark the samples from which each MAG was obtained. (B) Data and organization as in panel A but not considering the fraction of MAG covered for samples used to bin each MAG prior to ordering. Download FIG S2, PDF file, 0.3 MB. Copyright © 2022 Utter et al. 2022 Utter et al. https://creativecommons.org/licenses/by/4.0/ This content is distributed under the terms of the Creative Commons Attribution 4.0 International license.

10.1128/mbio.02414-22.5 FIG S3 Distribution of MAGs recruiting reads over at least 50% of their length. Each of the 390 vertical bars represents a different MAG, ranked in descending order, and the height of the bar shows number of samples providing 50% detection (up to 81 samples). Download FIG S3, PDF file, 0.01 MB. Copyright © 2022 Utter et al. 2022 Utter et al. https://creativecommons.org/licenses/by/4.0/ This content is distributed under the terms of the Creative Commons Attribution 4.0 International license.

Having established that many MAGs were detected in multiple samples, we set out to characterize the population-level diversity and dynamics before assessing the dynamics within populations. The diversity of all MAG-defined populations was broadly stable over a 2-week period (Fig. 1C), suggesting that few or no major microbiome-level changes occurred within the time window analyzed. Similarly, among the 30 MAGs with the highest mean relative abundance, MAG relative abundance was generally stable within a mouth, in that most of these MAGs had lower coefficients of variation within each donor than across donors (Fig. 1D). Thus, populations detectable and abundant at one time point are likely to be detectable with similar abundances at future time points in samples taken from the same mouth, thereby allowing the investigation of subpopulation dynamics.

### Genome-wide fluctuations of subpopulations occur at daily timescales.

We investigated whether we could detect genome-wide sweeps within a MAG-defined population. DESMAN deconvolves a consensus or population genome, in this case each MAG, into haplotypes that represent major subpopulations (39). DESMAN uses SNV frequencies that belong to single-copy core genes (15) in a MAG to identify combinations of co-occurring SNVs over multiple metagenome samples. Operationally, each DESMAN haplotype corresponds to a different strain.

**FIG 2:**
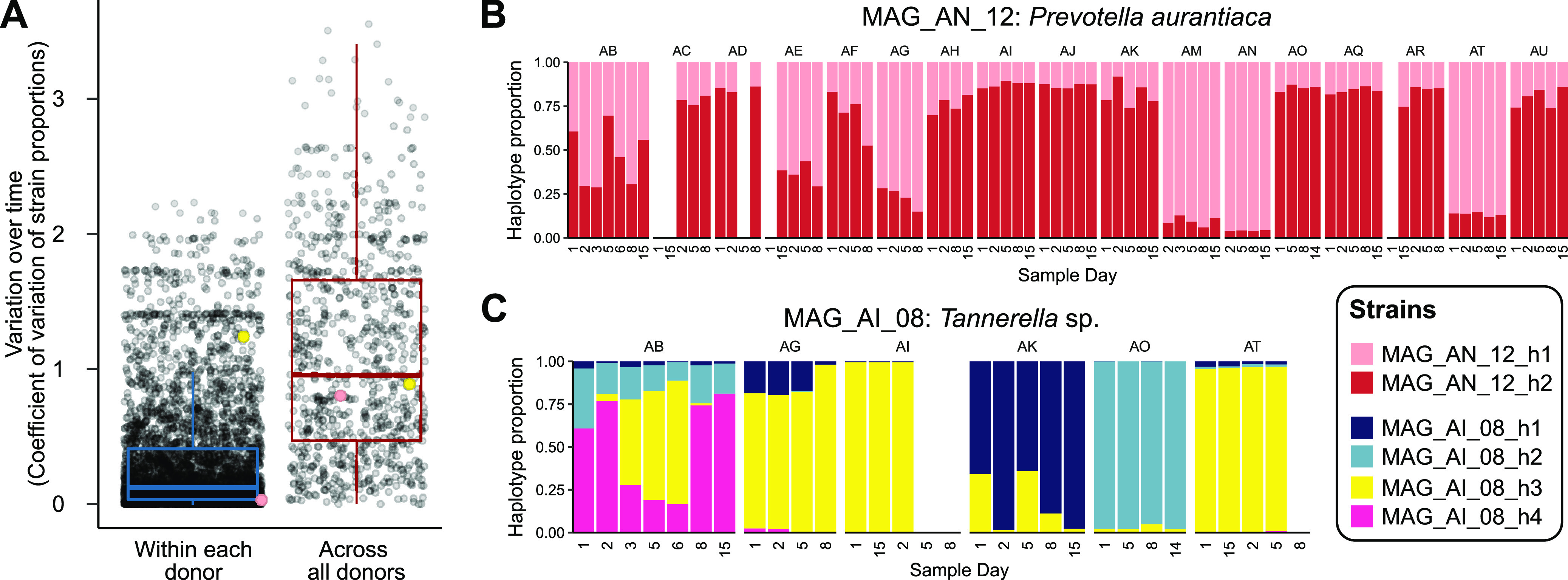
Strains are generally stable, but some rise to dominance over a period of days. (A) Coefficient of variation of strain proportions (proportion of parent MAG abundance) over time for each strain across all time points from all donors (red box plot) or within a single donor for each donor with that strain (blue box plot). Translucent points show individual strains’ coefficients of variation summarized by the box plots. (B) Example of stable strain dynamics. The colored bars show the proportions of the two strains relative to total MAG_AN_12 abundance. Strain proportions are not shown for samples in which the MAG or its core single nucleotide variants could not be detected. The points in panel A corresponding to MAG_AN_12_h1 are colored peach; the peach point in the within-donor category is for MAG_AN_12_h1 in donor AM. (C) Example of sweeping strain dynamics within MAG_AI_08. The layout and detection criteria are as for panel B. Yellow points in panel A correspond to MAG_AI_08_h3, with the yellow point in the within-donor category representing MAG_AI_08_h3 in donor AB.

All 390 MAGs were composed of one to five strains (median = 3) (Fig. S4), the majority of which were stable over time within a donor (Fig. 2). One example of a MAG with a stable strain composition is illustrated in Fig. 2B; all are presented in Fig. S5. For the example MAG_AN_12 in Fig. 2B, a 2.39-Mbp Prevotella aurantiaca MAG estimated to be 95% complete and 1.4% redundant, each of the two strains was detectable in all 17 mouths at similar proportions on day 15 and on day 1.

10.1128/mbio.02414-22.6 FIG S4 Histogram showing the distribution of the number of DESMAN-deconvolved strains across all MAGs. Download FIG S4, PDF file, 0.00 MB. Copyright © 2022 Utter et al. 2022 Utter et al. https://creativecommons.org/licenses/by/4.0/ This content is distributed under the terms of the Creative Commons Attribution 4.0 International license.

10.1128/mbio.02414-22.7 FIG S5 DESMAN haplotype decomposition for each MAG. The stacked, colored bars show the proportion (left *y* axis) of each haplotype (colors) in each sample, grouped by participant. The black line shows the coverage of the parent MAG (right *y* axis). Download FIG S5, PDF file, 2.2 MB. Copyright © 2022 Utter et al. 2022 Utter et al. https://creativecommons.org/licenses/by/4.0/ This content is distributed under the terms of the Creative Commons Attribution 4.0 International license.

Although strains were generally more stable within a donor than across donors (Fig. 2A), we also found examples of strains that exchanged dominance over a period of days, e.g., those from MAG_AI_08, a 2.59-Mbp *Tannerella* sp. MAG with 95% completion and 0.7% redundancy (Fig. 2C). For example, on day 2, h4 accounted for approximately 75% of MAG_AI_08 coverage from volunteer AB, while h3 represented around 5% of the population. One day later, the population was 50% h3 with just over 25% h4. By day 6, h3 was almost 75% of the population, and then by day 8, the relationship reverted, and h4 was at 75% abundance with h3 barely detected. These strain-level dynamics are consistent with the ecotype hypothesis, which predicts that distinct genotypes exist within a population that change in abundance over time.

While some strains dropped below the detection limit, some strains persisted but at low abundance, indicative of a partial sweep where a particular strain rose to dominance without driving other variants to extinction, e.g., h3 and h4 of MAG_AI_08 in volunteer AB on day 6 (Fig. 2C). This suggests that oral populations maintain a standing diversity of strain-level genotypes that may rise to dominance at a later time. The standing diversity of genotypes making up a given population, i.e., the alpha diversity of strains, differs both among populations and among mouths (Fig. 2; Fig. S5) although sampling and sequencing depth may affect the resolution.

**FIG 3:**
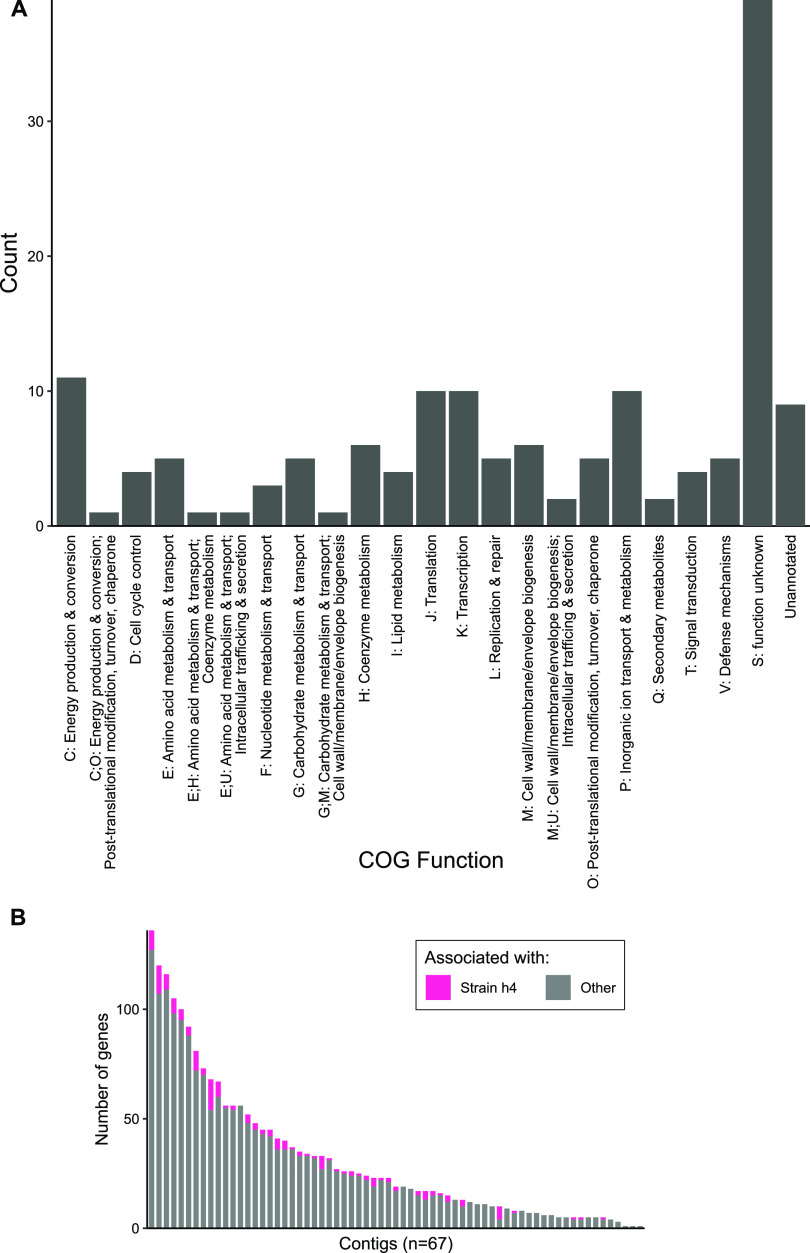
Genes associated with MAG_AI_08_h4. (A) Histogram of COG categories for the 155 genes associated with MAG_AI_08_h4. (B) The number of genes per MAG_AI_08 contig associated with h4 (pink) or not (gray) reveals that strain-associated genes are well distributed across the MAG.

Correlating gene coverage to strain proportional abundance within a MAG allows inference into what genes are associated with a dynamic strain, which in turn allows us to evaluate whether a strain matches expectations for an ecotype. If a strain represents an ecotype, i.e., a distinct ecoevolutionary unit, then it should have a unique set of genes that enable its success in a distinct niche. We define strain-associated genes to be those tightly correlated (Pearson *r* > 0.8) with a strain’s abundance across all samples but not so to other strains (*r* < 0.2). Applying this threshold to an exemplar strain (h4) of MAG_AI_08 revealed 155 genes associated specifically with this strain (Table S2; Fig. S6). These genes encompass a broad variety of functions from numerous COG (clusters of orthologous groups) categories, including metabolic genes such as a *susD*-like gene and an arginine decarboxylase gene, further supporting the hypothesis that this strain may represent an ecotype with a unique complement of metabolic genes that allow success in a specific niche (Fig. 3A; Table S2). We also investigated whether the strain-associated genes were distributed throughout the genome, indicative of multiple independent events, or distributed as a single contiguous block, indicative of a single gene transfer event such as a phage or mobile element. Strain-associated genes in the case of MAG_AI_08_h4 were scattered across most of the 67 contigs, with few strain-associated genes per contig (Fig. 3B). Altogether, the genes associated with this highly dynamic strain matched expectations for an ecotype.

10.1128/mbio.02414-22.2 TABLE S2 Predicted functions for the 155 genes associated with MAG_AI_08_h4. Download Table S2, XLSX file, 0.03 MB. Copyright © 2022 Utter et al. 2022 Utter et al. https://creativecommons.org/licenses/by/4.0/ This content is distributed under the terms of the Creative Commons Attribution 4.0 International license.

10.1128/mbio.02414-22.8 FIG S6 Correlation of gene coverage versus strain relative abundance for all genes (*x* axis) and strains (colors) in MAG_AI_08. Genes are arranged to group similar correlation patterns, not any chromosomal order. Download FIG S6, PDF file, 0.1 MB. Copyright © 2022 Utter et al. 2022 Utter et al. https://creativecommons.org/licenses/by/4.0/ This content is distributed under the terms of the Creative Commons Attribution 4.0 International license.

### Gene-level dynamics are observed but rarely become fixed.

Having detected genome-wide sweeps within the oral microbiota, we investigated the extent to which specific genes swept through populations. Operationally, we define gene sweeps as occasions where individual genes change in frequency within populations independent of their surrounding genomes. To detect such cases, we compared the coverage of each gene to the other genes in each MAG. The degree of correlation between genes can inform about their representation within a population, since genes on the same chromosome should correlate in coverage perfectly. Any deviation in coverage correlation between gene pairs thus reflects the existence of other factors that break this simple relationship. Such factors include nonspecific mapping to highly conserved genes in distantly related bacteria (e.g., housekeeping genes in organisms from different ecological niches), copy number variation, and a history of horizontal gene transfer with another population(s). These different factors may not necessarily be active within our 2-week study, but we searched for gene-specific changes to provide context for the genome-specific observations.

**FIG 4:**
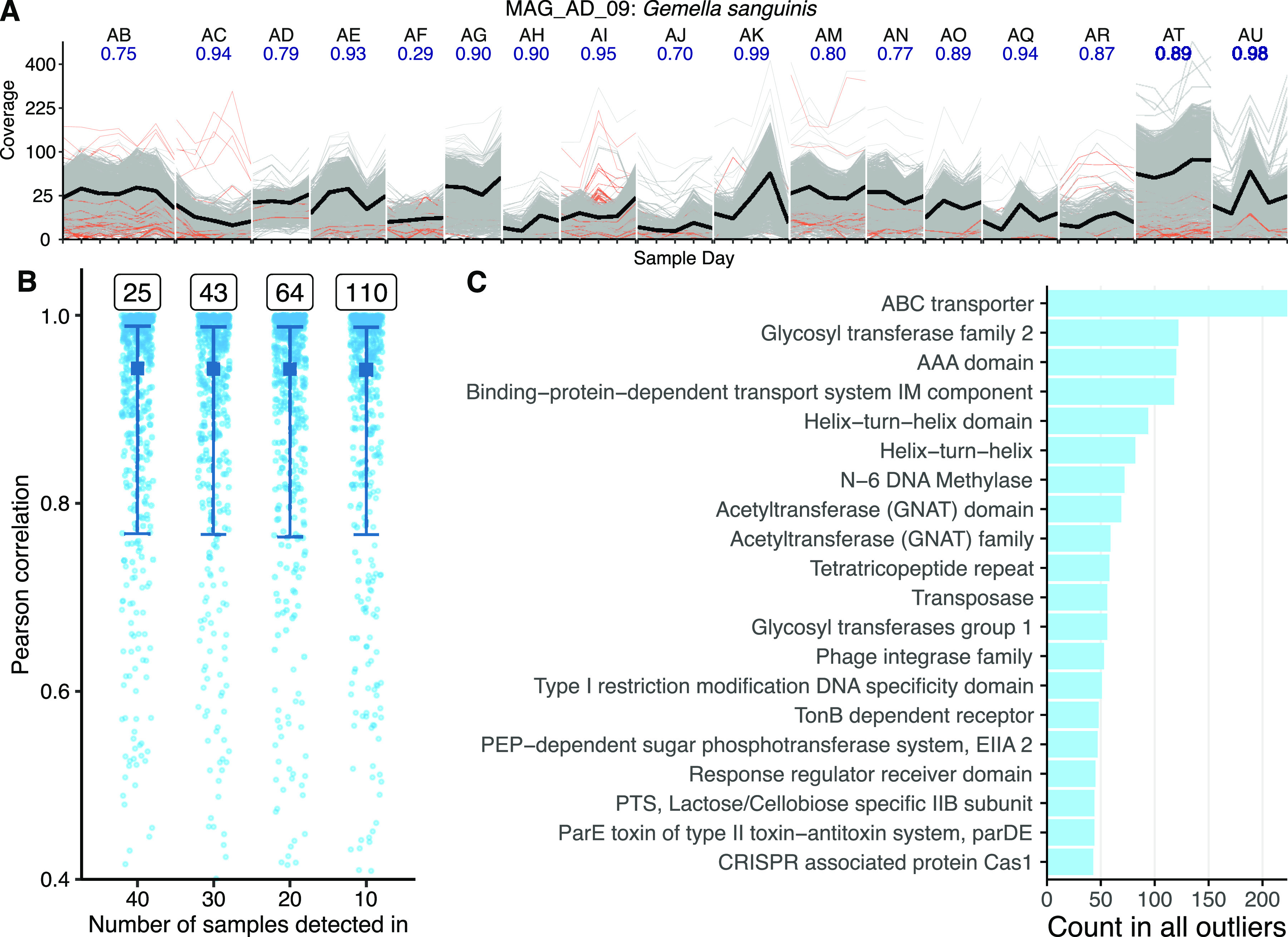
Most genes in a population are tightly correlated within a single mouth over time. (A) Coverage of each gene (thin gray lines) in an example MAG, MAG_AD_09, across time points. The thick black line shows the mean MAG coverage. The blue number beneath the participant label reports the median Pearson correlation for all gene pairs. Lines representing genes with a correlation coefficient less than 0.2 are orange. (B) Summary of all pairwise gene correlations. All possible gene x gene pairs were correlated by donor for MAGs with at least 75% of their nucleotides covered in 40, 30, 20, and 10 samples. The text box above each distribution number of MAGs meeting each detection criterion shown on the *x* axis. Dots show a random subsample of all gene pairs; the full distribution is summarized by the median (thick center box) and error bars (25% and 75% quantiles). (C) The top 20 predicted Pfam functions are shown for the genes with correlation coefficients less than 0.2 in the 40-sample detection threshold set (25 MAGs). Unannotated genes are omitted. IM, inner membrane; PEP, phosphoenolpyruvate; PTS, phosphotransferase system.

Gene abundances within an exemplar population tended to be stable and tightly correlated over days to weeks, with a few exceptions (Fig. 4A). We focused on MAG_AD_09, a Gemella sanguinis MAG, as an exemplar MAG due to its being detected across all donors (Table S1). The majority of genes within MAG_AD_09 were tightly correlated in most donors, with median Pearson correlation coefficients greater than 0.8 for 12 of 17 donors. However, a small fraction of genes were substantially less correlated, operationally defined as a correlation coefficient less than 0.2 (Fig. 4A, orange lines). However, relatively few of these decoupled genes maintained disproportionately high or low frequencies for the remainder of the sampling period.

This trend of overall tight gene correlation within mouths held true for all MAGs meeting various detection criteria (Fig. 4B). Like MAG_AD_09, the median correlation coefficient was above 0.80 despite increasing the number of MAGs from 25 to 110 (MAGs detected in 40 of 80 samples versus 10 of 80 samples). However, genes were less tightly coupled across donors than within donors (Fig. S7). Thus, we infer that most genes are tightly coupled over short timescales within oral bacterial populations, with a few exceptions.

10.1128/mbio.02414-22.9 FIG S7 Full distribution of the pairwise gene correlations summarized in Fig. 4. For each MAG with 75% of the genome covered in 5, 10, 20, 30, and 40 samples (subpanel rows), the Pearson correlation (*x* axis) over time for all gene pairs was calculated mouth by mouth (blue distributions; only for ≥20 sample detections) or across mouths (orange). A random subset of 1 million correlations were subsampled from each set and used to calculate the probability density distribution (*y* axis). The vertical line in each distribution marks the median correlation coefficient, and the translucent color spans the second to the third quartile. Download FIG S7, PDF file, 0.05 MB. Copyright © 2022 Utter et al. 2022 Utter et al. https://creativecommons.org/licenses/by/4.0/ This content is distributed under the terms of the Creative Commons Attribution 4.0 International license.

Inspection of the 20 most frequent Pfam functions in the set of decoupled genes revealed an enrichment for phage and mobile elements (Fig. 4C). This result could stem from a combination of nonspecific mapping from related phage and mobile elements in different populations, or it could implicate mobile elements as mechanisms for short-term change. Intriguingly, genes annotated as Cas1, a CRISPR-associated protein expected to play a role in defense against foreign DNA, ranked highly in the set of decoupled genes.

## DISCUSSION

Previous studies of the oral microbiome have introduced and supported the concept of “dynamic stability,” where populations defined by 16S rRNA ASVs fluctuate on daily timescales around a mean that is stable over time (19, 20, 40). Metagenomic surveys have dramatically increased the analytical resolution to reveal increasing levels of diversity and uncover the importance of strains to microbial ecology (41, 42). Our observations of fluctuating yet generally stable strains within populations are consistent with the concept of dynamic stability derived from amplicon studies but broaden the basis for that conclusion to entire genomes. For an individual tongue, most microbial populations were stably present over the 2-week sampling period. However, within these populations, some strains broke this pattern of intrapopulation stability, and specific genes were associated with these strains.

The observed patterns of gene and genome-wide dynamics distinguish the healthy oral microbiome from the healthy gut. Garud et al. (4) and Zhao et al. (24) reported that in the healthy human gut, gene sweeps or SNV accumulation is the most dominant process altering population genomes over 6-month timescales. However, each study applied different strain definitions, and the differences in sampling timescales preclude direct comparisons between studies. Others have highlighted the distinct differences between the oral and gut microbiomes in terms of composition, dynamics, and responsiveness to diet (43); here, we posit adding strain dynamics to the list. This difference could reflect differences in strain-level diversity between the oral and gut microbiomes, as we found oral strains with comparatively large genomic differences (up to hundreds of genes), while the gut microbiome literature reports strains typically differing by hundreds to thousands of SNVs or tens of genes (24, 44). Despite these differences, all studies agree on the existence and ecological relevance of multiple coexisting strains to each human microbiome.

Different definitions of “population” for bacteria have been given in the literature (13, 45). Populations have been defined by the existence of a unifying niche (6), sequence similarity thresholds obtained from marker gene or whole-genome alignments (46–48), measured or modeled recombination frequency (2, 49), or operationally based on geographic or seasonal distribution (50). Here, we use “population” to refer to a set of phylogenetically related lineages defined metagenomically. This definition has utility for our analyses, as it allowed us to use reference taxa to relate populations across mouths; e.g., we found that the Prevotella aurantiaca population defined by MAG_AN_12 in donor AM was dominated by a different strain than in donor AN. While each definition has strengths and weaknesses, the operational definition of a population as a MAG and the short reads that map to it is conceptually and practically useful; this definition is clear, it is approximate to the species concept, and it allows for the existence of subpopulations with distinct and potentially adaptive genetic features.

The coexistence of both gene and genome dynamics suggests that both processes work to shape oral bacterial populations, albeit differing in impact. While gene-specific and genome-wide sweeps fall under different concepts for species and population evolution (8), bacterial evolution is not constrained to the kinetics predicted by a single species concept (13). Our data suggest that both mechanisms of genomic change are relevant to the oral microbiome. We propose that the most impactful adaptive mode for the oral microbiome is genome-centric; ecotypes sweep to dominance in response to environmental changes. In addition, changes in gene frequency also occur, presenting a means of generating or modifying genetic diversity among ecotypes over longer timescales. While we suggest this model in the context of the oral microbiome, we anticipate that the broader application of high temporal and genomic resolution to other microbial systems will bring about a more complete understanding of microbial adaptation.

## MATERIALS AND METHODS

### Sample collection and library preparation.

We sampled the tongue dorsum from 19 self-described healthy human volunteers at days 1, 2, 5, 8, and 15 under institutional review board (IRB) oversight (Harvard University Committee on the Use of Human Subjects; IRB16-0367). Volunteers were between the ages of 20 and 29 years; 11 (58%) were female, 8 (42%) male. All participants were unrelated and not from the same household, and two participants (AB and AE) were in a relationship. Participants were sampled in the morning and were asked to abstain from eating or drinking anything besides water before sampling. Some donors were sampled at off days due to their availability (e.g., day 3 instead of day 2) or missed one of the 5 days; one donor was sampled 7 times (days 1, 2, 3, 5, 8, 13, 15). In total, 95 samples were self-collected by the volunteers with UV-sterilized disposable tongue scrapers (BreathRx; Philips, Stamford, CT). At each collection day, a sampling control was collected by means of a sterile scraper being opened and transferred to an empty collection tube. Samples were immediately flash-frozen in liquid nitrogen and stored at −80°C until DNA extraction.

DNA was extracted with Qiagen DNeasy PowerLyzer kits (Germantown, MD) for all samples and sampling controls and stored at −20°C until used for library preparation. A blank extraction (nothing added to extraction kit) was prepared along with each extraction batch as an extraction control. Only one control, a single extraction control, had detectable DNA with a TapeStation HS D1000 tape (Agilent), but the quantity was too low for sequencing at 0.01 ng/μL, and this control therefore was dropped. The 81 samples from 17 volunteers with DNA concentrations of >1 ng/μL were used to prepare metagenomic libraries with a Kapa DNA HyperPrep kit (Kapa, Wilmington, MA) according to the manufacturer’s recommendations. Input total DNA was sheared with a Covaris sonicator to an average fragment size of 600 bp. Libraries were quantified with a TapeStation (Agilent, Lexington, MA), pooled at equimolar concentrations, and size selected with a Pippin Prep system for 600- to 1,200-bp fragments.

### Sequence analysis and MAG construction.

The same library pool of 81 metagenomes was sequenced with 10 runs of Illumina NextSeq 550 with the high-output paired-end 150 bp kit, producing over 6 billion read pairs. Metagenomic short reads were quality controlled with illumina-utils (51) following quality recommendations by Minoche et al. (52). Human reads were discarded by mapping with bowtie2 (53) to the human genome (hg38), resulting in a total of 6,210,900,714 quality-controlled reads.

For each donor, the assembly program IDBA-UD used metagenomes from all of each donor’s time points to produce a single assembly, i.e., coassembly by donor. Each coassembly’s contigs were binned with CONCOCT (15) on the basis of tetranucleotide frequency and differential coverage of contigs across that coassembly’s samples and manually refined in anvi’o (23) using best practices for MAG refinement (37, 54). Manual refinement consisted of inspecting each bin to remove wrongly included contigs identified by outlier coverage or nucleotide variability in the metagenomes from which the contigs were assembled (16, 37). A set of 71 universal bacterial single-copy genes were used to assess genome completeness and redundancy (55, 56). Genes were called with Prodigal (57) with default parameters. Gene functions were predicted with Interproscan for Pfam and TIGRFAM annotations and with emapper v2 for eggNOG annotations, including NCBI COGs (58–63).

From these bins, MAGs were defined as bins that are less than 10% redundant (percentage of marker genes expected as a single copy occurring more than twice) and either have a size of at least 2 Mbp or are >80% complete (percentage of expected single-copy core genes detected). After refinement, the 17 coassemblies’ MAGs (*n* = 409) were pooled, and duplicate MAGs (e.g., substantially identical MAGs defined independently in different mouths) were identified as having ≥98% average nucleotide identity (ANI) over at least 75% of the smaller MAG’s length and a Pearson correlation coefficient of ≥0.85 for their coverage across all samples. Nineteen MAGs (5%) were identified as duplicate and discarded to retain a dereplicated set of 390 unique MAGs (Table S1). These criteria for MAG inclusion span both the high- and medium-quality draft genome categories set out by the Genomic Standards Consortium (55). Of 390 MAGs, 376 and 188 had redundancy estimates of ≤5% and ≤1%, respectively; therefore, combined with the manual curation based on the above parameters, the final 390 MAGs have a very low chance of including contaminant contigs from distant populations that could affect their interpretation.

MAGs were assigned taxonomy by GTDB-Tk v1.40 (64) using the Genome Taxonomy Database release 95 (38). While the final set of MAGs included representatives from the majority of common oral taxa (Table S1; Fig. S1), certain genera, namely, Streptococcus, *Veillonella*, Haemophilus, and *Neisseria*, did not assemble or bin well and were underrepresented. Their exclusion was likely due to the challenges posed to assembly algorithms by highly diverse populations (9, 65). Thus, our analyses focused on populations for which high-quality MAGs could be generated.

### Coverage-based definitions and metrics.

The final coverage was determined using Bowtie2 (53) to map the raw short reads to a single database containing all 390 nonredundant MAGs. Coverage for a collection of nucleotides, e.g., a gene or MAG, is reported as the average nucleotide’s coverage. All SNVs mapped by Bowtie2 were retained regardless of coverage depth to allow application-specific filtering. Detection for a collection of nucleotides, e.g., a gene or genome, is defined by a breadth-of-coverage criterion as the fraction of that collection’s nucleotides receiving any coverage at all. Relative abundance for a MAG in a sample is the percentage of reads recruited by a MAG relative to the total reads recruited by all MAGs.

### Haplotyping.

We used the SNV counts and DESMAN (39) following the developers’ recommendations to deconvolve each MAG into strain-level units. DESMAN attempts to split a consensus population genome (in this case, a MAG) into constitutive haplotypes by tracking SNVs in core genes that covary, using a set of 71 core genes found in a single copy in all known bacteria and archaea (15). DESMAN requires prior information on the expected number of haplotypes in the population. To estimate this, we decomposed each MAG into 1, 2, 3, 4, 5, 6, 8, 10, or 12 haplotypes for 500 iterations and plotted the mean posterior deviance for each MAG-and-haplotype combination. The optimal number of haplotypes for each MAG was determined by SNV uncertainty below 10% (39) and a posterior deviance step of >10%.

### Detecting outlier genes.

To estimate the linkage of genes within a population, we also correlated coverage of gene pairs for all possible pairs in a MAG. For each MAG, we correlated gene pairs using only the samples from mouths covering at least 75% of that MAG’s nucleotides for 40, 30, 20, 10, and 5 samples. Correlations were determined mouth by mouth, iterating mouth by mouth using all samples from one mouth at a time, and across all samples from all donors meeting the detection criteria. Mouth-by-mouth correlations were performed only for MAGs detected in 40, 30, 20, and 10 samples due to the lack of change and the computationally infeasible number of pairwise correlations for all MAGs meeting the detection criteria in 5 samples. To identify functions between MAGs, we used the same MAG-level detection thresholds but instead correlated each gene’s coverage to the MAG’s coverage.

### MAG and strain naming conventions.

MAGs are named in the format MAG_[DONOR]_[NUMBER], where [DONOR] is an alphabetical identifier for the donor from which that MAG was assembled and [NUMBER] is a unique number to distinguish multiple MAGs assembled from the same donor. DESMAN haplotypes (strains) append “_h[NUMBER]” to the MAG designation; e.g., MAG_AI_08_h4 is the fourth strain of the eighth MAG from donor AI.

### Ethics approval and consent to participate.

All samples were collected following IRB approval and oversight (Harvard University Committee on the Use of Human Subjects; IRB16-0367).

### Data availability.

The raw metagenomic data are publicly available at NIH NCBI BioProject PRJNA879058 under BioSample accession numbers SAMN30789053 to SAMN30789143. Analyzed data in the form of anvi’o databases can be found at https://doi.org/10.6084/m9.figshare.6025748. A reproducible methods document at https://dutter.github.io/projects/diversity_dynamics provides the complete bioinformatic workflow, including the code used for all analyses, with a full explanation.
