# Supplementary figures and images for: Genome-Centric Dynamics Shape the Diversity of Oral Bacterial Populations
Source: mBio. 2022 Oct 10;13(6):e02414-22. doi: 10.1128/mbio.02414-22 (PMC9765137; doi:10.1128/mbio.02414-22)

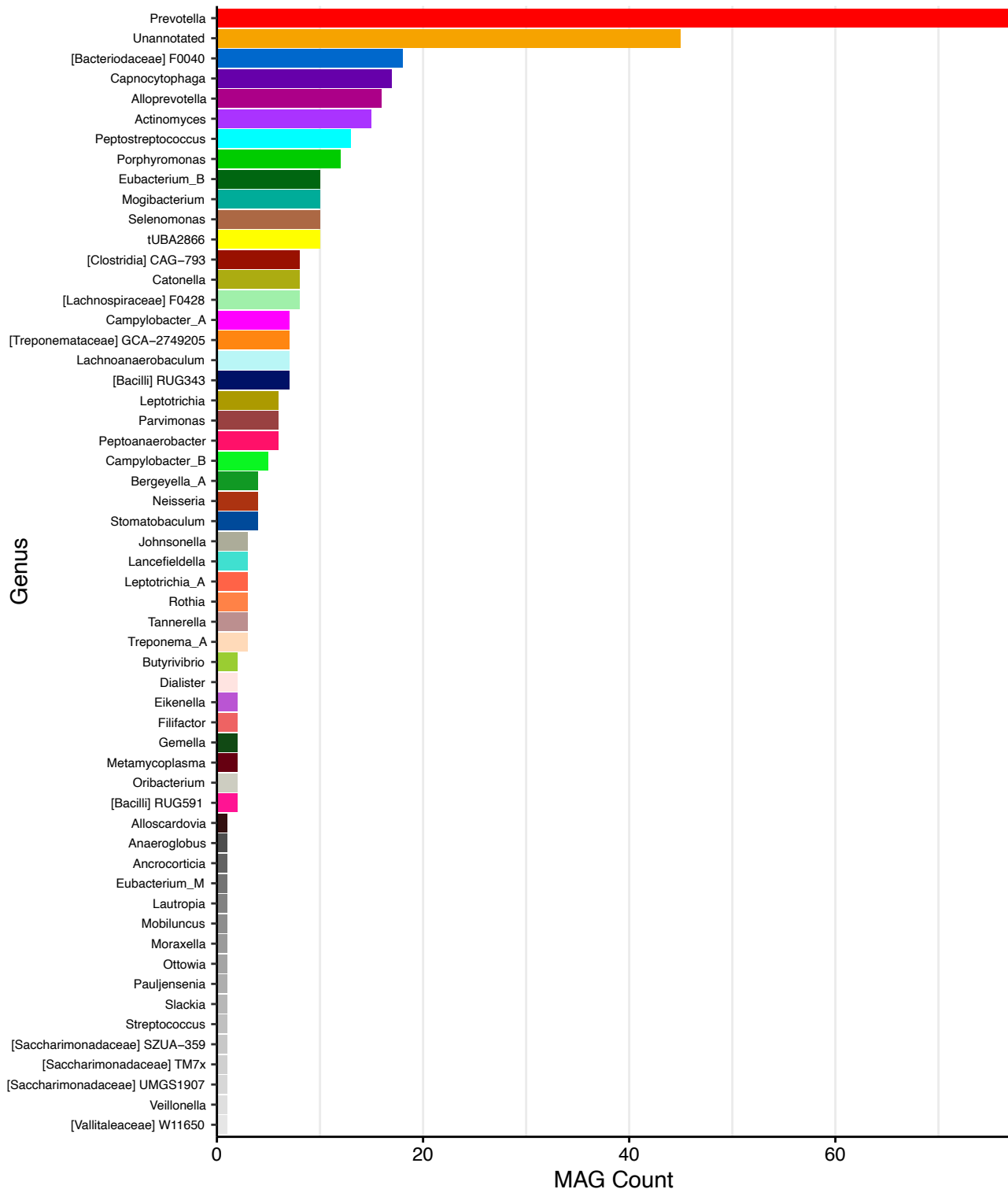

Supplement: FIG S1 [file mbio.02414-22-s0002.pdf]

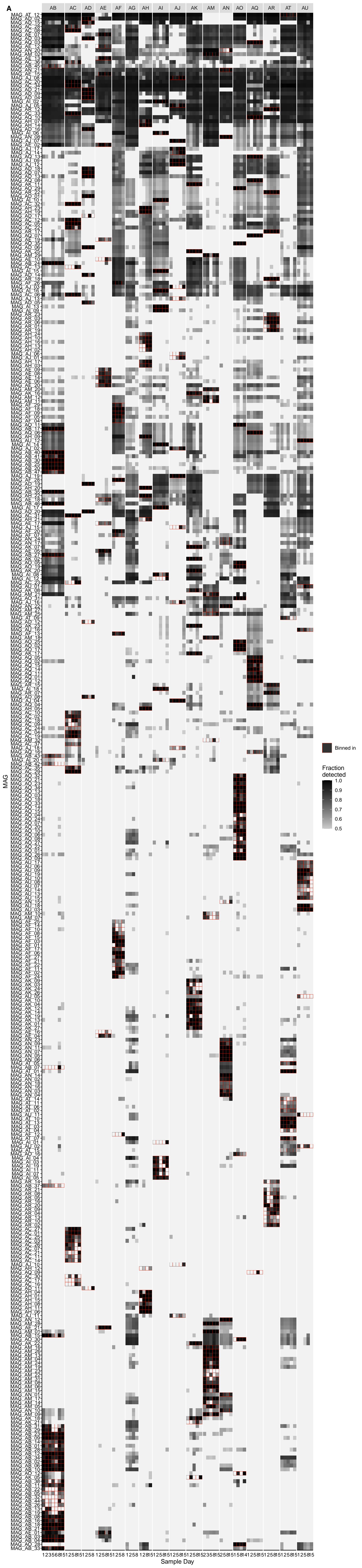

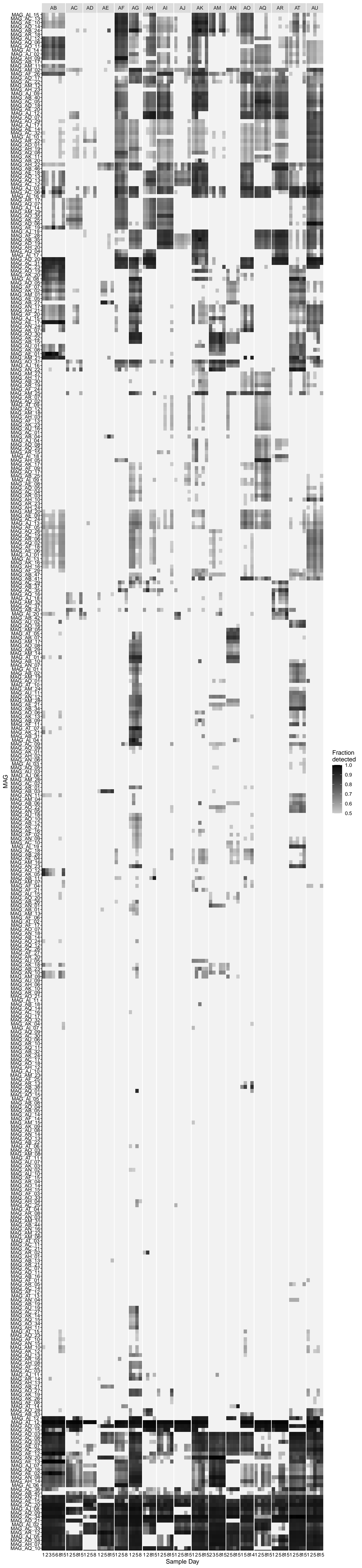

Supplement: FIG S2 [file mbio.02414-22-s0004.pdf]

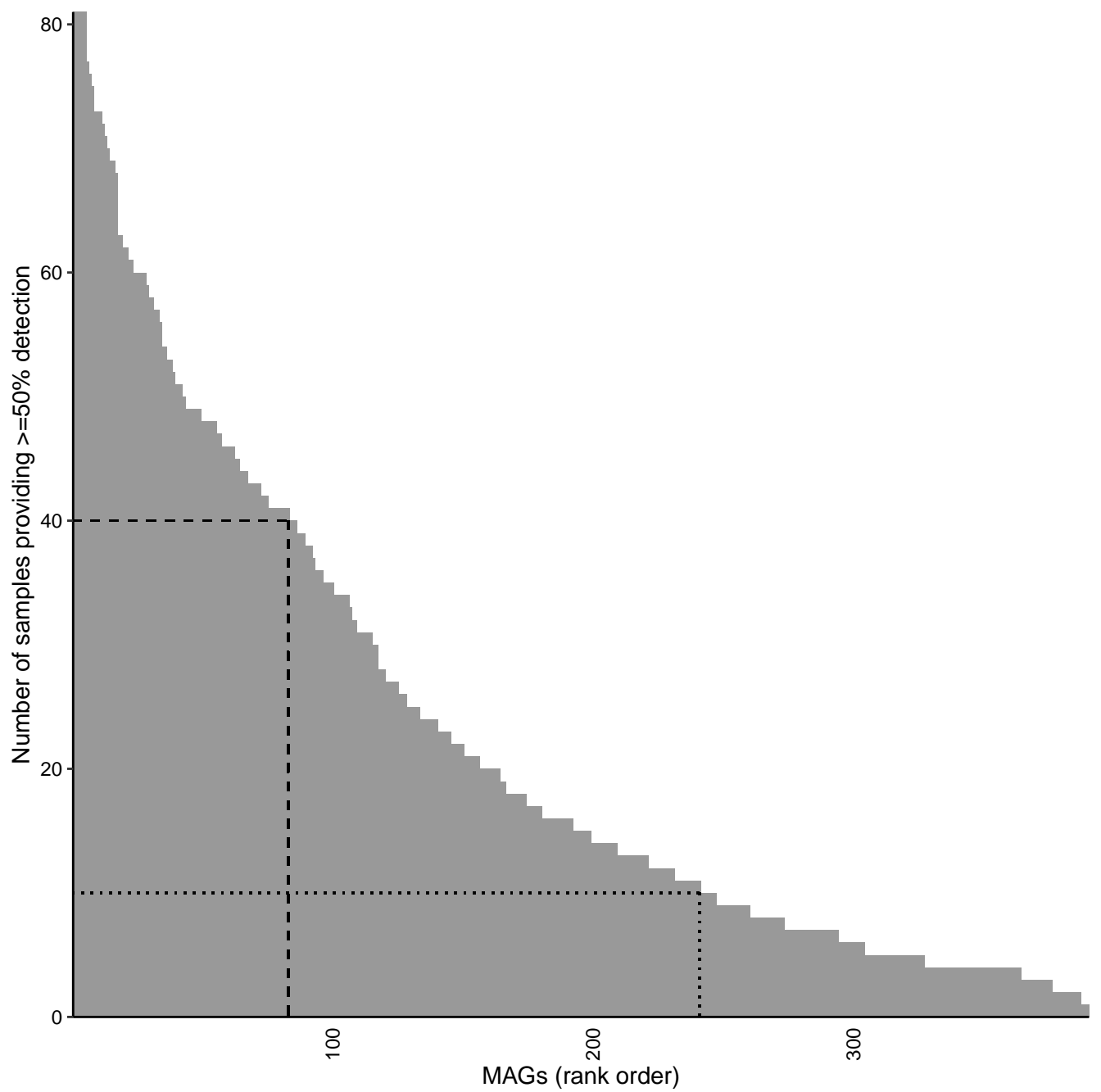

Supplement: FIG S3 [file mbio.02414-22-s0005.pdf]

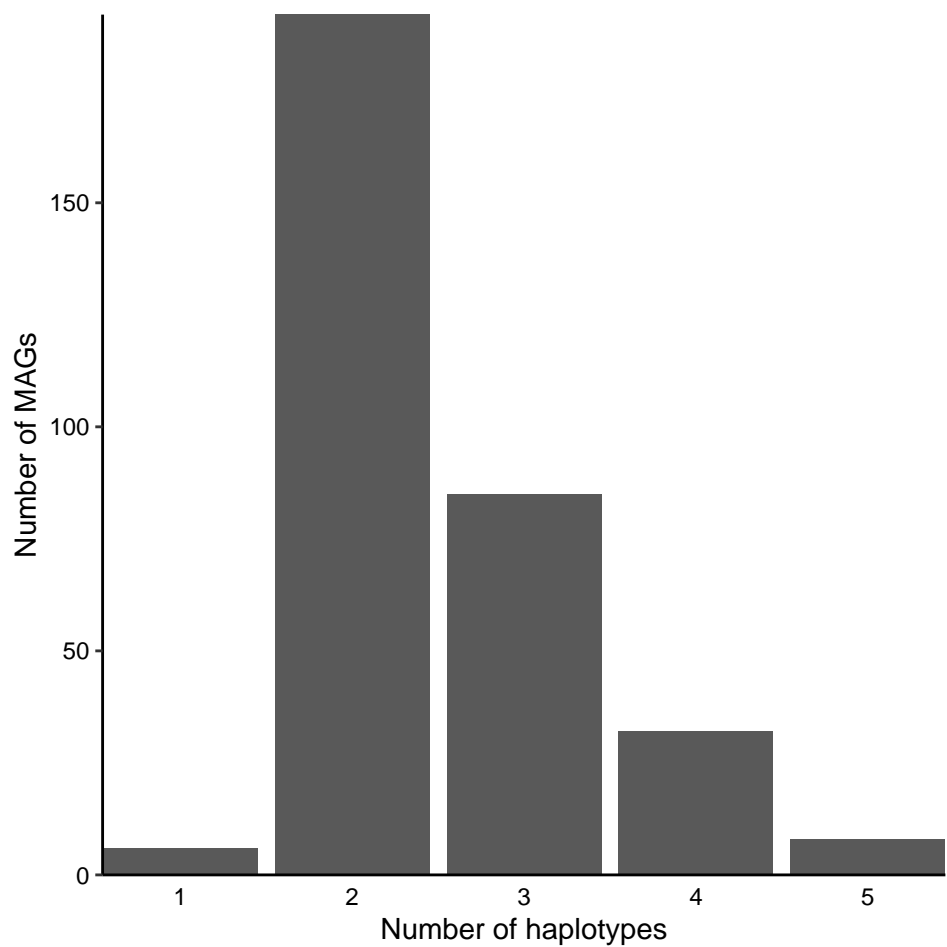

Supplement: FIG S4 [file mbio.02414-22-s0006.pdf]

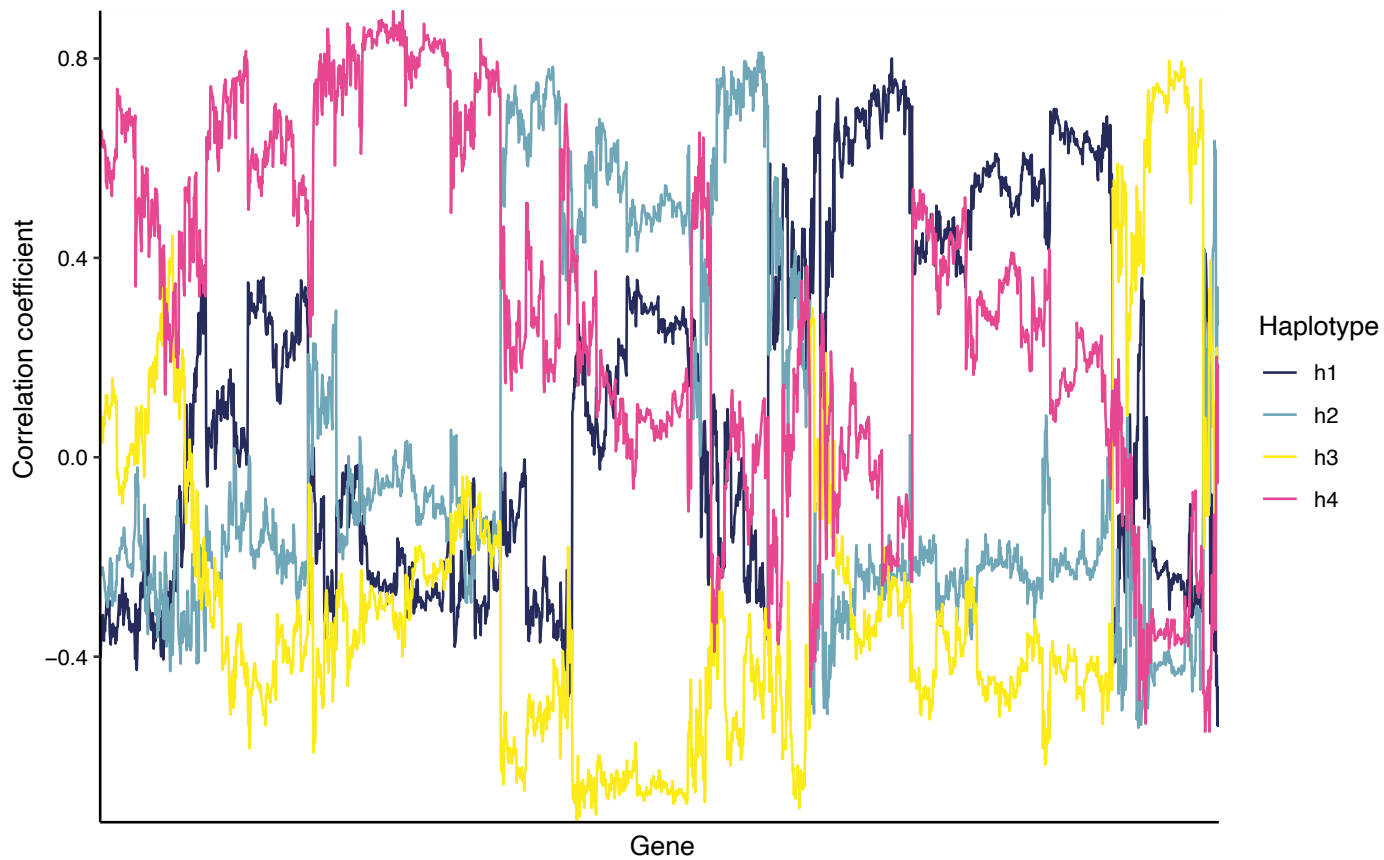

Supplement: FIG S6 [file mbio.02414-22-s0008.pdf]

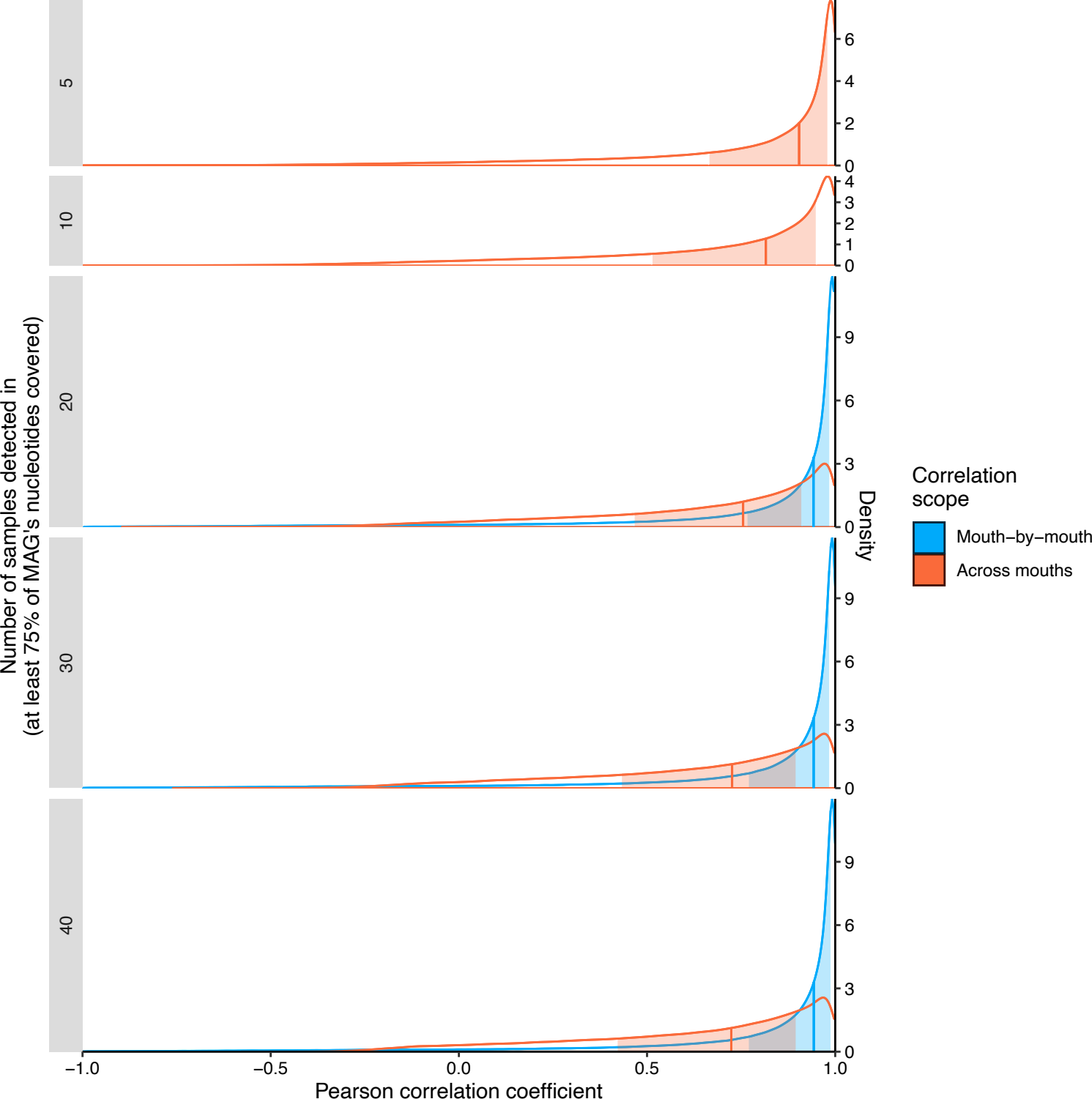

Supplement: FIG S7 [file mbio.02414-22-s0009.pdf]
